# Nutrition in Health and Disease

**Published:** 1882-12

**Authors:** 


### NUTRITION IN HEALTH AND IN DISEASE.

[Translated by the Editor from the *Journal D'Hygiene.*]

IN examining the functions of digestion in health and disease it will be well to notice the principal phenomena.

Difficult digestion and nutrition—often neither recognized nor treated—are the cause of a great number of functional and organic diseases which attack more especially the kidneys and the liver and which prepare the way for a multitude of morbid conditions.

Apart and in addition to the general symptoms of difficult digestion generally recognized and studied, the examination, physical, microscopic and chemical, of the urine gives the most delicate and the most certain indication of the condition of the digestive and nutritive functions. If these last are defective, a few hours after partaking of food the urine generally contains morbid salts and often becomes turbid on cooling. The presence of these salts under the circumstances is proof of an imperfect digestion of food and an imperfect nutrition is the necessary consequence. This is why their existence is a danger for the organism and a danger which is not less real on account of being more or less distant. This clinical fact offers to the physician a guide, the surest, the most tangible for the treatment, hygienic, dietetic and medical, of the malady. The patient himself, when he knows the significance and the importance of the urinary deposits, finds in the trouble which these deposits occasion in his urine when it is cooled an easy guide to follow in order to regulate his food and the ordinary conditions of his life.

Many persons are unconscious invalids and suffer and die through the silent work of a defective nutrition, although the morbid changes often precede the malady and could generally be modified or combated with success.

A defective nutrition in the first years of life frequently escaping the observation of those who have charge of children often gives the key to problems which one meets in practice. Parents enjoying good health, young, endowed with good constitutions, living in the midst of favorable social conditions, sometimes have children who, seemingly in the enjoyment of good health from birth, unexpectedly sicken and die of consumption or other disease contracted while growing up.

We have seen parents, in good health, lose one after another of their children from consumption, without any tangible cause. We are convinced that the real cause of these accidents has been most frequently an error in the regimen, the nourishment, and in defective nutrition during childhood.

The analysis of the vital functions would be incomplete if we failed to recognize the mysterious vital influence which governs all organic modifications.

It is this vital principle, transmitted from one living being to another, which produces this singular phenomenon ; that with a physical organization very similar, one animal lives and grows by consuming grain, a second by eating herbs, a third by eating meat, and a fourth by eating all three things. They may explain this fact by a "vitality inherent ;" but the explanation is not entirely satisfactory. All our knowledge in chemistry, in anatomy and physiology is powerless to make us penetrate this great mystery.

The all-powerful Author of all things has endowed every living creature with inherent power to live and to reproduce an organism resembling his own ; but the reason of variations in this vital power can only be found in His sovereign will. With our limited knowledge, the nature of this vital power is totally incomprehensible. The force of inherent vitality is not only different in each kind of animal or of plant, but in each member of each kind. We are able, by applying the laws developed by science and by experience, to estimate its extent in each individual, but only in very narrow limits.

In health, as in life, "the prize of the combat is not always to the strongest, nor the race to the swiftest." Many whose parents and ancestors possessed good constitutions show no vital force, and succumb if they are subjected to the test or attacked by disease. Others, on the contrary, who have not good antecedents from the side of their parents, and whose health is not good, pass through trials, mental and physical, the most severe, and often attain an advance age. With them "the battle of life" is so vigorous, so tenacious, that it resists influences the most unfavorable. Children can be neglected and badly nourished ; men can be exposed to fatigue, to distress moral, to bad air, to all sorts of maladies, and, thanks to the force of their inherent vital power, they resist all morbid influences, or, if they succumb for a time, they afterward raise themselves up and replace their feet upon the shore of life.

Among those that belong to this class may be counted habitual drunkards who attain old age ; those who support during seventy years diseases and physical suffering ; those who live long years in a sickly country ; in countries where mortal diseases reign. The soldier who escapes the balls of the enemy, in twenty campaigns, is an example of this fact ; the lawyer, who arrives to his dignities after fifty years of brain work, and of bodily inaction.

The vital principle must have been exceptionally puissant, the attachment to life exceedingly strong, from the moment the individual breathed for the first time. There have been exceptions to general rules which govern health and life ; these exceptions explain themselves by the intensity of the vital power, which we recognize by its results, but which we have not the power to comprehend nor always to foresee.

It is one of the most beneficent favors of Providence that such exceptions can be produced. It is the hope of longevity given to the sick and feeble. No science, no human genius is able to estimate, surely, the vital power inherent in an animated being, even if he is borne down by disease ; if no organ indispensable to life is irremediably attacked ; the vitality of the invalid can often raise him up, overcome the malady, and permit him to attain an age accorded to but few men.

